# DYSMORPHIC features and adult short stature: possible clinical markers of KBG syndrome

**DOI:** 10.1186/s13052-021-00961-5

**Published:** 2021-01-25

**Authors:** Davide Mattei, Paolo Cavarzere, Rossella Gaudino, Franco Antoniazzi, Giorgio Piacentini

**Affiliations:** 1 Department of Pediatrics, University Hospital of Verona, Verona, Italy; 2 Pediatric Clinic, Department Surgical Sciences, Dentistry, Gynecology and Pediatrics, University of Verona, Verona, Italy; 3 Regional Center for the diagnosis and treatment of children and adolescents rare skeletal disorders. Pediatric Clinic, Department of Surgical Sciences, Dentistry, Gynecology and Pediatrics, University of Verona, Verona, Italy

**Keywords:** Short stature, Dysmorphic feature, KBG syndrome, NGS approach, Case report

## Abstract

**Background:**

Growth monitoring is an essential part of primary health care in children and short stature is frequently regarded as a relatively early sign of poor health. The association of short stature and dysmorphic features should always lead to exclude an underlying syndromic disorder.

**Case presentation:**

We report the case of an Indian school-aged boy with dysmorphic features, intellectual disability and a clinical history characterized by seizures and hearing problems. Although his height was always included in the normal range for age and sex throughout childhood, he presented a short near-adult stature in relation to his mid-parent sex-adjusted target height. This is probably due to a rapidly progressive pubertal development.

**Conclusions:**

In the presence of characteristic dysmorphic features, intellectual disability, seizures and hearing problems, KBG syndrome should always be considered. This emergent condition presents a wide spectrum of clinical phenotypes and is often associated with adult short stature.

## Introduction

Human growth is a very complex phenomenon regulated by genetic, hormonal, nutritional and environmental factors. Although it continues from the embryonic life to puberty and early adulthood, it is a dynamic process such that normal height at one specific moment does not exclude the possibility that short stature (SS) appears later, because of innate or acquired growth disruptors [1]. Thus, childhood growth monitoring is an essential part of primary health care in children, and SS must be regarded as a relatively early sign of poor health [2]. SS can have a wide variety of causes. Although the GH-IGF axis has a central role with specific actions on growth, numerous genes are involved in the control of stature [3]. Nonetheless, these genes explain only a small fraction of the phenotypic variation in height. Multiple disorders characterized by prenatal and/or postnatal onset growth failure have been described and, among these, there are many new syndromes unravelled in the last decades [4, 5]. Consequently, the aim of the endocrinology evaluation for a child with SS is to investigate the possible causes of his condition with particular focus on possible syndromic disorders, especially if dysmorphic features are present.

## Case presentation

Here we describe the clinical history of an Indian school-aged boy. He was born at term in India by emergency C-section, due to prolonged labour after an uneventful pregnancy. Birth weight was 2500 g (− 1.95 standard deviations score [SDS]) and birth length 49 cm (− 0.50 SDS), for the evaluation of neonatal measures we used Bertino Neonatal Anthropometric Charts [6]*.* Parents were not related and had a normal stature (mid-parent sex-adjusted target height 174.5 cm). No noteworthy disease was reported in the family history. In the neonatal period, no complications were described. He was able to walk alone only at 18 months old. The family moved to Italy when he was 3 years old without major clinical problems. When he was 6 years old, during the first year of school, learning difficulties emerged. Thus, he was referred to an infant neuropsychiatrist who submitted him to some investigations. In particular, karyotype was normal and the fragile X syndrome was excluded. A brain MRI showed bilateral diffuse subependymal heterotopia, partially empty sella and hypoplastic anterior pituitary. After these findings, he was referred to a pediatric endocrinology evaluation, which was not performed.

When he was 7 years old, he underwent adenoidectomy for recurrent middle ear infections. Subsequently, he continued to present numerous episodes of otitis media, which led to bilateral conductive moderate degree hearing loss. At 9 years of age, after a CT scan of petrous bones detected chronic erosive otitis and a bilateral tympanic membrane perforation, he underwent bilateral myringoplasty. At 11 years old, he was hospitalized for a first episode of seizures. Epilepsy was diagnosed and a specific treatment was started.

**Fig. 1:**
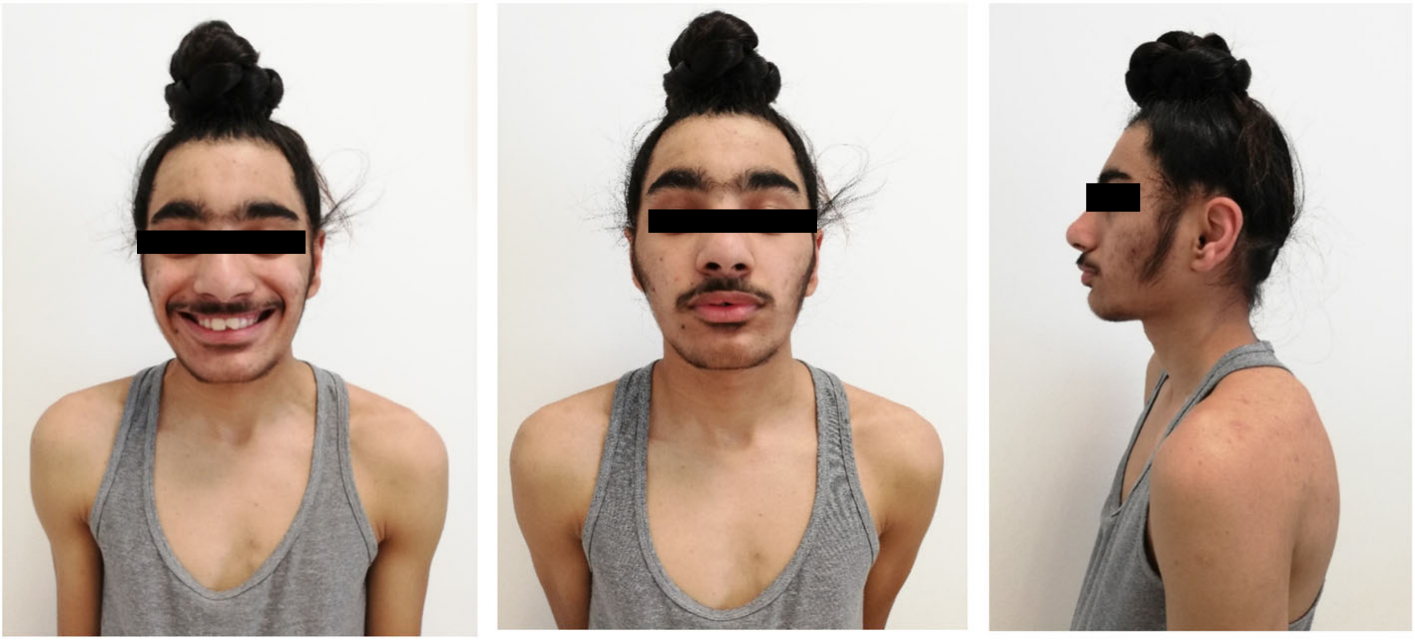
Pictures of our patient are represented

**Fig. 2:**
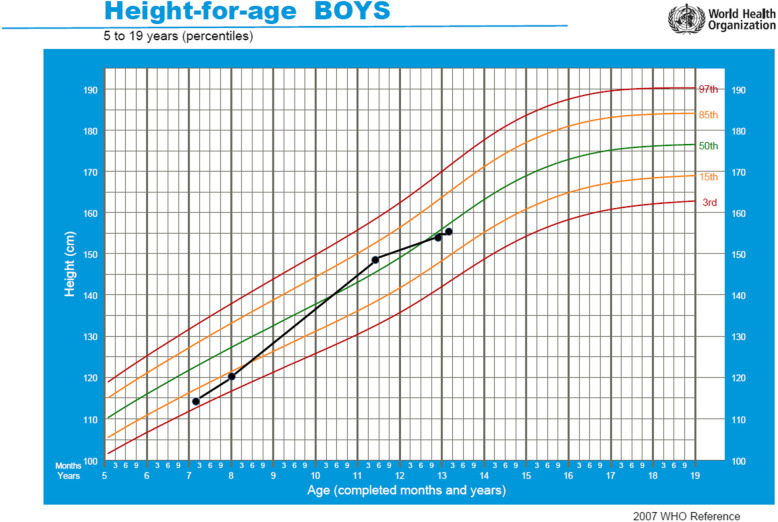
The growth chart of our patient is represented

He came to our attention for the first time in May 2017, when he was 11 years and 5 months old. His height was 147.9 cm (0.18 SDS) and his weight 34.5 kg (− 0.87 SDS), SDS were derived from Cacciari Italian cross-sectional growth charts [7]. At our clinical examination, he appeared in good general conditions, with normal body proportions, but a moderate psychomotor delay emerged. Besides, some syndromic features were noticed: macrodontia of the upper central incisors, synophrys, widely spaced eyes, bushy eyebrows and bulbous nose (Fig. 1). Genital examination revealed a Ph 3 and a testicular volume of 10 mL bilaterally. A true bilateral gynecomastia was observed. Bone age was advanced over chronological age by ∼2 years. IGF1 level was normal (57.03 nmol/L, 1.10 SDS for age and sex). Thyroid function was normal. Furthermore, we performed a GnRH-analogue test that confirmed a normal activation of hypothalamus-pituitary-gonads axis and an ACTH test that excluded late onset congenital adrenal hyperplasia as cause of bone age advancement. A new brain MRI confirmed the previous one. Despite we recommended a follow up every 6 months, he came back to our attention only 18 months later, when height was 154 cm (− 0.29 SDS), weight 39.1 kg (− 1.10 SDS) and pubertal development was complete. Thyroid, adrenal and gonadal functions remained normal. Bone age was 16 years and 6 months (versus a chronological age of 13 years). His height was in the normal range for chronological age; however, considering his complete pubertal development and his bone age, his prediction of adult height according to Byley and Pinneau tables was only 157.5 cm (Fig. 2). Considering his dysmorphic features and his SS, we decided to further investigate the case in the suspect of Cornelia De Lange syndrome. Next-generation sequencing (NGS) panel analysis was performed and a de novo variant heterozygous in exon 9 on ANKRD11 gene was detected (NC_000016.9:g89351043_89351047del, NM_013275:c.1903_1907del; with a predicted protein change p.Lys635GInfs*26). This mutation is causative of KBG syndrome [8, 9]. Therefore, clinical and anamnestic characteristics of our patient led us to diagnose KBG syndrome, which is an emergent condition with a wide spectrum of clinical phenotypes, often associated with adult short stature. Once the definitive diagnosis was obtained, in order to define the extent of disease and needs of our patient, he was subjected to complete and multidisciplinary evaluations aimed at identifying the most common anomalies present in the KBG syndrome. In particular, cardiological and ocular abnormalities were excluded; a class II malocclusion was evidenced and a large right tympanic perforation was diagnosed, for which surgery was necessary.

The study was conducted in compliance with the terms of the Helsinki II Declaration. Written informed consent was obtained from the patient’s parents for publication of this case report and any accompanying images. In our country, namely Italy, this type of clinical study does not require Institutional Review Board/Institutional Ethics Committee approval to publish the results.

## Discussion and conclusions

KBG syndrome is a rare autosomal dominant disorder with multiple congenital anomalies and intellectual disability. The disorder was first described in 1975 as a new “malformation/retardation syndrome” in three families; consequently, its name derives from the initials of the last names of three original families diagnosed with this syndrome [10]. KBG syndrome was initially thought to be quite rare; however, it is likely underdiagnosed because many of its features are often mild and non-specific, and none of them are a prerequisite for the diagnosis [11]. To date, almost 200 subjects affected by the syndrome have been reported [12]. Although several familiar cases have been described, most of the affected individuals have novel de novo mutations. It is worth mentioning that there is significant variability in the clinical findings, even between affected members of the same family and frequently, a mildly affected mother is recognized only after a typically affected son is diagnosed [13].

KBG syndrome should be suspected in an individual with developmental delay/cognitive impairment or significant behavioral issues in association with other features such as distinctive craniofacial characteristics, SS and skeletal anomalies. Eight major clinical criteria were initially defined: macrodontia of the upper central permanent incisors (which is reported in 85–95% of affected individuals), characteristic facial appearance, hand anomalies, neurological involvement, delayed bone age, costovertebral anomalies, SS, and the presence of a first-degree relative with KBG syndrome [8]. Afterwards, delayed bone age and costovertebral anomalies were suggested to be removed, and macrodontia was not considered a mandatory criterion any longer [12–16]. Minor features that can further help to establish a clinical diagnosis are otitis media and hearing impairment, seizures, cryptorchidism, feeding problems, palatal insufficiency, and delayed anterior fontanelle closure [14]. However, no consensus clinical diagnostic criteria for KBG syndrome have yet been published.

Our patient presented with a typical subset of clinical (such as macrodontia of the upper central incisors, synophrys, widely spaced eyes, bushy eyebrows and bulbous nose) and anamnestic (such as neuro-developmental delay, seizures and otitis media) characteristics of KBG syndrome; nevertheless, we initially hypothesized a Cornelia de Lange syndrome. The clinical signs that made us suspect this syndrome were SS, intellectual disability, seizures, craniofacial abnormalities, in particular synophyrys and hirsutism. Mild Cornelia de Lange syndrome, in fact, shares many findings with KBG syndrome; however, patients tend to have smaller head circumference and greater degrees of intellectual disability [17, 18]. The clinical presentation of Cornelia de Lange syndrome has a very wide spectrum and can closely resemble other genetic disorders, especially in non-classical phenotypes. Therefore, genetic testing is recommended to make a definitive diagnose and rule out other genetic disorders. The first-line molecular diagnostic approach should be NGS-based screening focused on NIPBL, SMC1A, SMC3, RAD21, BRD4, HDAC8, and ANKRD11 genes. According to the first consensus statement about the diagnosis and the management of Cornelia de Lange syndrome, this specific panel is considered the most effective way of detecting causative variants in all the genes known to cause Cornelia de Lange syndrome [19]. This approach allowed us to diagnose our patient as affected by KBG syndrome. In particular, as previous mentioned, a de novo variant heterozygous in exon 9 on ANKRD11 gene was detected (NC_000016.9:g89351043_89351047del, NM_013275:c.1903_1907del; with a predicted protein change p.Lys635GInfs*26). Generally, single nucleotide variants within ANKRD11 gene account for approximately 83% of pathogenic variants and larger copy number variants (mostly deletions) involving ANKRD11 gene detectable by chromosomal microarray account for approximately 17% of cases [15, 18, 20].

The definitive diagnosis of KBG syndrome is rarely achieved before the upper permanent central incisors have erupted at age 7–8 years [13]. Nevertheless, focusing on all the dysmorphic features is key to anticipate the age of definitive diagnosis. In this way, patients could benefit from early multidisciplinary interventions allowing better outcomes, especially with regards to growth and neurocognitive performances. Overall, with appropriate management, the prognosis of the syndrome is good [12].

Our patient was sent to our attention after the execution of brain MRI, which detected subependymal grey matter heterotopia, partially empty hypoplastic sella turcica and hypoplastic anterior pituitary gland tissue. Although the exact frequency of brain malformations is not known, several brain abnormalities have been reported in various cohorts of affected individuals [8, 18, 21]. When he was 11 years old, he developed epilepsy characterized by tonic-clonic seizures associated to EEG abnormalities; this symptom has been reported in about 50% of affected individuals, with age of onset variable between infancy and teenage years [14, 22]. In addition to these neurological abnormalities, he presented with history of learning difficulties, that required addition aid to attend mainstream classes, and development delay, for which he had been followed up for a long time. More than 90% of patients with KBG syndrome have some degree of developmental delay, especially in speech [20, 22]. Besides neurologic features, another typical anamnestic element was the hearing impairment. Our patient suffered from recurrent episodes of otitis media throughout the childhood that ended to perforate eardrums, requiring bilateral myringoplasty. About 30% of affected individuals report hearing problems, with conductive loss being the most frequent [18].

Variable skeletal anomalies have been reported in 75% of individuals affected by KBG syndrome. The most frequent findings are costovertebral anomalies, such as cervical ribs, abnormal vertebral shape, end plate abnormalities, posterior fusion defects, or spina bifida occulta [15]. Other anomalies include a short and webbed neck, abnormal ribs, brachydactyly, clinodactyly, scoliosis, cardiac defects [20], ocular findings [13], skin and hair abnormalities [14]. Our patient did not presented any of the aforementioned anomalies, except for a clinical hirsutism that could be due to his ethnic group.

SS is very common among these patients, being found in 40–77% of affected individuals [20, 23]. At birth, auxological parameters are usually normal, as in our patient, and only later SS becomes evident. Preliminary evidence suggests that growth hormone treatment may increase the height potential of affected subjects [23, 24]. When our patient came to our attention for the first time, his height was in the normal range for sex and age (0.18 SDS). He presented clinical signs of intermediate pubertal development, confirmed also by serum levels of testosterone and an advanced bone age of 2 years. After 18 months, his bone age had further advanced, widening the gap between bone age and chronological age, and, thus, considerably affecting his final height prediction. While SS and delayed bone age are frequently related to KBG syndrome, rapid pubertal development and advanced bone age are uncommon [12, 18]. By observing his growth curve, reconstructed thanks to the data noted by his paediatrician, what emerged was that his stature was always included between the normal range for age and sex throughout childhood, with an abnormal increase in stature between 8 and 11 years. This might be due to an unidentified precocious puberty, which has been already described in children with KBG syndrome [20]. A premature activation of puberty may reasonably explain the rapid advancement in bone maturation and be one of the main causes of his short final stature. If precocious puberty had been promptly identified and treated with GnRH-analogous, the slowdown in puberty development would have perhaps allowed an increase in our patient’s growth potential [12]. Considering that for male adults affected by KBG syndrome is reported a mean stature of 153.6 cm [13], we could say that the current height of our patient is included in the normal range. However, it is reduced in relation to his mid-parent sex-adjusted target height. Furthermore, contrary to what happens in the majority of individuals affected by the syndrome, our patient did not show a spontaneous catch-up growth between childhood and adulthood [23].

This case report offers some insights. First, the link between dysmorphic features, SS, and intellectual disability confirms to be a common red flag for syndromic disorder. We recommend to investigate the clinical hypothesis of KBG when a child presents dysmorphic features, even of mild degree, regardless of the presence of SS, because normal height at one specific time during childhood does not exclude the possibility of SS occurring later in development. Second, the NGS approach reveals to be extremely useful in the diagnostic process. In presence of clinical suspicion of syndromic disorder, we suggest proceeding as soon as possible with pediatric genetic evaluation and/or genetic analysis with NGS approach, in order to reach the correct diagnosis and to establish the most appropriate treatment and follow up.

In conclusion, in the presence of dysmorphic features, intellectual disability, and a clinical history characterized by seizures and hearing problems, KBG syndrome should always be considered and excluded. This emergent condition presents a wide spectrum of clinical phenotypes and is often associated with adult SS.

## Data Availability

Not applicable.

## Abbreviations

SS: Sort stature
SDS: Standard deviations score
MRI: Magnetic Resonance Imaging
NGS: Next generation sequencing
